# Influence of advanced age of maternal grandmothers on Down syndrome

**DOI:** 10.1186/1471-2350-7-4

**Published:** 2006-01-14

**Authors:** Suttur S Malini, Nallur B Ramachandra

**Affiliations:** 1 Human Genetics Laboratory, Department of Studies in Zoology, University of Mysore, Manasagangotri, Mysore 570 006, India

## Abstract

**Background:**

Down syndrome (DS) is the most common chromosomal anomaly associated with mental retardation. This is due to the occurrence of free trisomy 21 (92–95%), mosaic trisomy 21 (2–4%) and translocation (3–4%). Advanced maternal age is a well documented risk factor for maternal meiotic nondisjunction. In India three children with DS are born every hour and more DS children are given birth to by young age mothers than by advanced age mothers. Therefore, detailed analysis of the families with DS is needed to find out other possible causative factors for nondisjunction.

**Methods:**

We investigated 69 families of cytogenetically confirmed DS children and constructed pedigrees of these families. We also studied 200 randomly selected families belonging to different religions as controls. Statistical analysis was carried out using logistic regression.

**Results:**

Out of the 69 DS cases studied, 67 were free trisomy 21, two cases were mosaic trisomy 21 and there were none with translocation. The number of DS births was greater for the young age mothers compared with the advanced age mothers. It has also been recorded that young age mothers (18 to 29 years) born to their mothers at the age 30 years and above produced as high as 91.3% of children with DS. The logistic regression of case- control study of DS children revealed that the odds ratio of age of grandmother was significant when all the four variables were used once at a time. However, the effect of age of mother and father was smaller than the effect of age of maternal grandmother. Therefore, for every year of advancement of age of the maternal grandmother, the risk (odds) of birth of DS baby increases by 30%.

**Conclusion:**

Besides the known risk factors, mother's age, father's age, the age of the maternal grandmother at the time of birth of the mother is a risk factor for the occurrence of Down syndrome.

## Background

India represents the largest human diversity, consisting of 4,635 culturally and anthropologically well defined populations with very little gene flow between them. Myriads of castes, subcastes and tribes, high degree of endogamy and consanguinity in various sects along with a population of more than one billion, India provides an excellent opportunity for birth defect investigations.

DS is the most common and readily identifiable chromosomal anomaly associated with mental retardation and occurs in one out of 600 live births [1,2]. Studies revealed three genetic mechanisms to cause DS viz: free trisomy 21 (92–95%), mosaic trisomy 21 (2–4%) and translocation (3–4%) [3]. In all high birth frequency of DS studies, trisomy 21 has been a subject of interest to the clinicians and researchers due to its complexity in phenotype expression. Eventhough there are over 50 clinical symptoms of DS, it is rare to find all or most of them in one person [4]. Inheritance of DS is still not completely understood. However, earlier workers strongly advocated that the advanced maternal age is a major risk factor for trisomy 21 [5-11]. The likelihood that a woman under 25 and 30 years who becomes pregnant will have a baby with DS is less than 1 in 1,400 and 1,000 respectively. Chance of having a baby with DS increases to 1 in 350 for women who become pregnant at age 35 and continues to increase as the woman ages, so that by age 42, and by age 49, the chance is 1 in 60 and 1 in 12 respectively [5]. On the contrary there are reports that 80% of DS babies are born to young women of age less than 30 years [2,12].

Nondisjunction occurs when chromosomes fail to segregate during meiosis and is the major cause of pregnancy wastage and mental retardation in humans. At least in 5% of all clinically recognized human pregnancies, meiotic segregation errors give rise to zygotes with the wrong number of chromosomes. The nondisjunction error is more frequent in first meiotic division (80%) rather than second meiotic division (20%) [13]. The polymorphic microsatellites have revealed that Trisomy 21 is due to nondisjunction of 90% of the maternal and 10% of paternal chromosome [14].

DS is the major cause of mental retardation because a large number of DS children are born in diverse populations of India. DS has not been examined extensively. However, information on risk factors for DS among babies born to young women is limited. The occurrence of DS in other parts of the world is ranging from 0.9–2/1000 live births. In India the prevalence of DS is still not clear because of limited work. Survey in a few places indicates the prevalence to be in the range of 0.81–1.2/1000 live births [15-17]. It has been reported that the mean maternal age of the DS children is around 30 years in Hyderabad, Mumbai and Punjab [18-22].

Bittles and Glasson [23] stated that "until our understanding of the mechanisms that underlie chromosomal nondisjunction advances to the point that we can effectively prevent this crucial causal event in the production of trisomy 21, the number of individuals with DS in the population is likely to increase". However, current trends indicate that, unless trisomy 21 conceptions are prevented, fetuses will be conceived and infants continue to be born with DS. The ultimate goal of research on DS should be to improve the lives of people with DS and their families. Much remains to be done to reach this goal [24]. In the present investigation we quantify the effect of maternal, paternal and grandmaternal age as well as consanguineous marriages on the occurrence of DS.

## Methods

### DS cases

We have ascertained 69 DS cases from different hospitals of Mysore city for a period of three years. All the cases were cytogenetically confirmed using standard methods and grouped as to the type karyotype. The age of the patient ranged from newborn to 15 years. An informed consent was obtained from the parents.

### Control population

We have randomly selected two hundred families belonging to different religions as well as different localities in and around Mysore city, South India. To generate case-control-dataset, 69 cases of DS and one randomly selected child from each of the 200 control families were used.

### Establishment of Genetic Register and Pedigree

Genetic register was maintained to collect the complete clinical assessment of the proband, information pertaining to age, sex, religion, caste, habits, health status; medical, family and reproductive histories of the parents, and parental age at the time of conception. Age of the mother and father at the birth of the child as well as age of the grandmother at birth of the mother was also recorded for all the families under study. With these informations, the pedigree of the families under study was constructed.

### Statistical analysis

The logistic regression was performed using the software, SPSS version 10.0 to record the effect of the variables. Case-control-status was used as dependent variable and age of mother, father and grandmother as well as an indicator of consanguineous marriage as co-variates. Results are reported as odds- ratios from models with one variable at a time as well from a model with all four variables simultaneously.

## Results

Most of the DS cases had common symptoms like mental retardation, broad short hand, small mouth, short neck, abnormal ear lobe, and delayed developmental skills. The number of DS children showing simian crease was less (31%) and none of the cases encountered traits like brush field spots. Among the DS children 53% were females. The cytogenetic studies of the 69 DS cases revealed that 67 were free trisomy 21, two cases were mosaic trisomy 21 and none with translocation.

**Figure 1:**
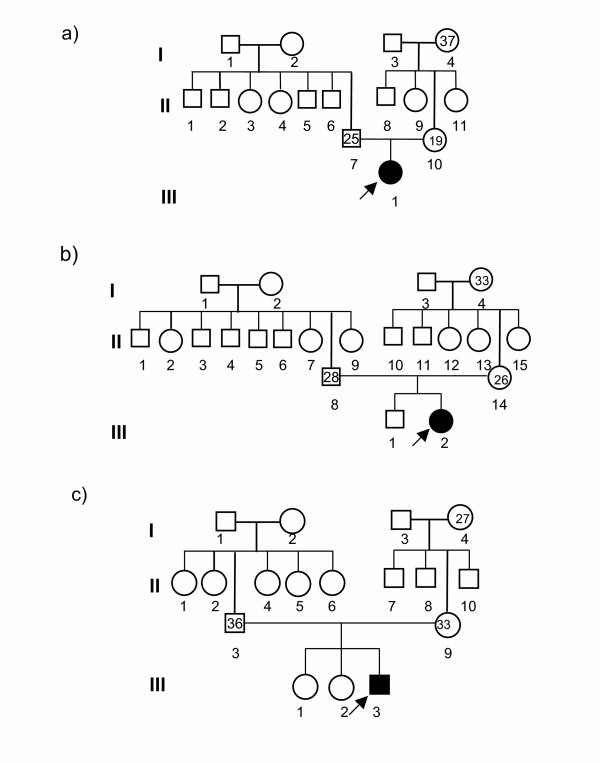
Pedigree of families of 19 years young mother (a) and 26 years young mother (b) and 33 years advanced age mother (c) with DS children. The Roman number in the left side of the figure indicates the number of generations. The Arabic number below the symbol denotes the number of individuals in that generation. The number inside the symbol of grandmother represents the age when she gave birth to the mother of DS. The number inside the symbol of father and mother in the 2^nd ^generation indicates their age when they gave birth to DS child. The arrow directed to the shaded symbol in the 3^rd ^generation represents the DS child. These are the representative pedigrees out of 69 DS families.

Table 1 presents the age of parents and number of children born in 69 DS and 200 control families. Figure 1 illustrates the pedigree of families of 19 years young mother (a), 26 years young mother (b) and 33 years advanced age mother (c) with DS children. Perusal of the pedigree indicates the relationship of age of mother and maternal grandmother in the family. The pedigrees show the order of birth of mother and father and also DS children. The information of parental age was used to relate the age of the grandmother.

**Table 1:** Distribution of parental age and number of children born in 69 Down syndrome cases and 200 control families.

Age range (in years)	No. of children at different age range born to					
	
	Mother		Father		Maternal Grandmother	
	
	Downs	Controls	Downs	Controls	Downs	Controls
18–24	34	312	01	59	03	548
25–29	18	133	20	160	03	204
30–34	12	56	29	208	37	96
35–40	05	12	13	65	24	06
> = 41	-	08	06	29	02	04

Total	69	521	69	521	69	858

Table 2 shows the results of the logistic regression of case – control study of DS children. The analyses were done at all combinations to establish specific relationship of grandmother's age with other variables. The 95% confidence intervals for the effect of age of mother, age of father and consanguineous marriage were lower than the age of maternal grandmother. The odds ratios were significant when all the four variables were used one at a time. When the age of father and mother were considered as covariates there was no significant difference in the odds ratios. At the four variables levels only grandmother's age showed significant difference in the odds ratios. These analyses once again support the fact that advanced age of the grandmother is a risk factor.

**Table 2:** Logistic regression analyses of case – control study of Down syndrome children (c.i = confidence intervals).

Variable	Univariate	Multiple
	Odds ratio (95% c.i.)	Odds ratio (95% c.i.)
Mother (per year)	1.14* (1.07;1.20)	1.07 (0.94;1.23)
Father (per year)	1.14* (1.07;1.20)	1.14 (0.99;1.32)
Maternal grandmother (per year)	1.25* (1.19;1.32)	1.30* (1.22;1.39)
Consanguineous marriage	2.55* (1.35;4.81)	1.84 (0.75;4.50)

*p < 0.001

## Discussion

Out of the 69 cases of DS studied, 67 of them were found to be free trisomy 21; two mosaic trisomy 21 and none with translocation. It has been reported that most of the translocation DS cases were also born to younger mothers [25,26]. Such translocation DS cases were not found in our study. The findings also revealed that 75% of DS children were born to young mothers whose age ranged from 18–29 years. In the control group remarkable difference in the number of children born at different age range of mothers and fathers establishes that more children were born to the young mothers (18–24 years) and father of advanced age (30–35 years). The number of children born to young mothers is also more when compared to fathers of the similar age. This is probably due to women getting married at young age (18–29 years) and produces more children in Indian families. This kind of situation is not found in western families. The age distribution between the mother as well as the father of the DS cases and controls indicate that both maternal and paternal age has no decisive influence for the manifestation of DS.

Understanding of the basic mechanism behind the maternal age effect is lacking. However, there are a very few earlier reports indicating the influence of grandmaternal age, on the risk of their grandchild being born with DS [27-29]. In the present study, the logistic regression analysis using all the four covariates have shown that when these covariates were considered together the effects of mother's age, father's age and consanguineous marriage were diluted but still of clinical relevance, albeit not statistically significant. However, the effect of age of the maternal grandmother was not diluted, showing an increase in odds by 30% per extra year. If we look into the pedigree of these families, it is clear that whenever the daughter was born to aged mother the chances of this daughter giving birth to DS children are increased.

To account for the maternal age effect and to explain susceptible exchange configurations associated with nondisjunction of chromosome 21, Lamb et al., [1], hypothesized a two-hit model. The first hit is unrelated to maternal age and involves the formation of a susceptible tetrad resulting from a specific exchange pattern established prenataly during meiosis I. The second hit involves some age-related disturbance of the meiotic process. Such a disturbance might involve any part of the meiotic apparatus [1]. In addition to this, an altered recombination pattern along nondisjoined chromosomes is the first molecular correlate identified for nondisjunction in humans [30]. Jeffreys et al [31] have also demonstrated that *Drosophila *oocytes exhibit significant age-dependent meiotic nondisjunction wherein achiasmate chromosomes become vulnerable to nondisjunction as *Drosophila *oocytes age.

Taking into the cognizance of these informations, we propose that advanced age of grandmother is responsible to bring disturbance in the meiosis of her daughter when the grandmother conceived. At the advanced age the grandmother's reproductive system may fails to make the essential proteins like spindle associated proteins, factors responsible for resting of oocyte, chiasma-binding proteins, DNA repair enzymes, etc. which are needed for proper meiotic segregation in the germ cells of her daughter. The non-availability or non-functioning of proteins leads to impairment in the meiotic process, which in turn results in nondisjunction of chromosome 21 in the oocyte of the daughter. This event takes place during the embryogenesis of the mothers of the DS children when she was in grandmother's womb. It is also possible that recombination is reduced in the oocytes, which brings about the nondisjunction of chromosome 21. Therefore, DS not only depends on the age of the mother but also on the age of the maternal grandmother which results in nondisjunction of chromosome 21.

The information pertaining to the age of grandmother, father and mother, as well as consanguinity were recorded during the data collection by interviewing the family members. However, birth records of a few individuals were not available. Although controls were selected randomly in different locations of Mysore including all the religions, this selection cannot be absolute because some of the families did not agree for investigation. These findings can be applied to the families with larger progenies in India or elsewhere. Further investigations are needed to understand the importance of chiasma formation and factors responsible for the proper meiotic segregation of germ cells during the foetal development in the advanced age mothers.

Further, the publication of the finished sequence of human chromosome 21 and the annotation of genes within it have provided the resources for characterizing each gene and demonstrating its potential relevance to the DS phenotype [32]. Hence, it can be surmised as Patterson and Costa [24] put it "because of the unprecedented experimental and theoretical tools that are available today, it is not unreasonable to speculate that even the complicated cognitive disabilities that are associated with DS might be amenable to therapeutic interventions designed to help people with DS to maximize their potential".

## Conclusion

Age of the maternal grandmother at the time of birth of the mother is a risk factor for the occurrence of Down syndrome, as is the age of the mother, and the father and consanguineous marriage as previously established.

## Competing interests

The author(s) declare that they have no competing interests.

## Authors' contributions

SSM carried out the chromosomal studies, pedigree analysis, and drafted the manuscript. NBR conceived the idea, participated in the design of the study and statistical analysis and also coordinated the programme. Both the authors read and approved the final manuscript.

## Pre-publication history

The pre-publication history for this paper can be accessed here:


